# Effect of socio-demographic and health factors on the association between multimorbidity and acute care service use: population-based survey linked to health administrative data

**DOI:** 10.1186/s12913-020-06032-5

**Published:** 2021-01-13

**Authors:** Kathryn A. Fisher, Lauren E. Griffith, Andrea Gruneir, Ross Upshur, Richard Perez, Lindsay Favotto, Francis Nguyen, Maureen Markle-Reid, Jenny Ploeg

**Affiliations:** 1 School of Nursing, McMaster University, HSC 2J36, 1280 Main Street West, Hamilton, Ontario L8S 4K1 Canada; 2 Department of Health Research Methods, Evidence, and Impact, McMaster University, CRL Building, First Floor, 1280 Main Street West, Hamilton, Ontario L8S 4K1 Canada; 3 Department of Family Medicine, University of Alberta, 6-10 University TerraceEdmonton, AB T6G 2T4, Edmonton, Alberta T6G 2R3 Canada; 4 ICES, 2075 Bayview Ave, Toronto, Ontario M4N 3M5 Canada; 5 Division of Clinical Public Health, Dalla Lana School of Public Health, 155 College St. Room 690, Toronto, ON M5T 3M7 University of Toronto, Toronto, Ontario Canada; 6 Institute for Clinical Evaluative Sciences (ICES), McMaster University, HSC 4N43, 1280 Main Street West, Hamilton, Ontario L8S 4K1 Canada

**Keywords:** Acute care service use, Multimorbidity, Socio-demographic factors, Population-based cohort study, Older adults, Emergency department use, Hospital use

## Abstract

**Background:**

This study explores how socio-demographic and health factors shape the relationship between multimorbidity and one-year acute care service use (i.e., hospital, emergency department visits) in older adults in Ontario, Canada.

**Methods:**

We linked multiple cycles (2005–2006, 2007–2008, 2009–2010, 2011–2012) of the Canadian Community Health Survey (CCHS) to health administrative data to create a cohort of adults aged 65 and older. Administrative data were used to estimate one-year service use and to identify 12 chronic conditions used to measure multimorbidity. We examined the relationship between multimorbidity and service use stratified by a range of socio-demographic and health variables available from the CCHS. Logistic and Poisson regressions were used to explore the association between multimorbidity and service use and the role of socio-demographic factors in this relationship.

**Results:**

Of the 28,361 members of the study sample, 60% were between the ages of 65 and 74 years, 57% were female, 72% were non-immigrant, and over 75% lived in an urban area. Emergency department visits and hospitalizations consistently increased with the level of multimorbidity. This study did not find strong evidence of moderator or interaction effects across a range of socio-demographic factors. Stratified analyses revealed further patterns, with many being similar for both services – e.g., the odds ratios were higher at all levels of multimorbidity for men, older age groups, and those with lower household income. Rurality and immigrant status influenced emergency department use (higher in rural residents and non-immigrants) but not hospitalizations. Multimorbidity and the range of socio-demographic variables remained significant predictors of service use in the regressions.

**Conclusions:**

Strong evidence links multimorbidity with increased acute care service use. This study showed that a range of factors did not modify this relationship. Nevertheless, the factors were independently associated with acute care service use, pointing to modifiable risk factors that can be the focus of resource allocation and intervention design to reduce service use in those with multimorbidity. The study’s results suggest that optimizing acute care service use in older adults requires attention to both multimorbidity and social determinants, with programs that are multifactorial and integrated across the health and social service sectors.

**Supplementary Information:**

The online version contains supplementary material available at 10.1186/s12913-020-06032-5.

## Background

Multimorbidity – the coexistence of two or more chronic conditions in the same person –is highly prevalent in older adults. Studies from a range of settings and populations show an increasing prevalence of multimorbidity, with estimates ranging from 65 to 98% in those > 65 years of age [1–6]. Studies from around the world also report that the prevalence of multimorbidity is on the rise due to global aging, including increases in Canada, from 17.4 to 24.3% (27% increase) [7], the Netherlands, from 12.75 to 16.2% (12% increase) [8], and the U.S. from 22 to 30% (36% increase) [9]. Multimorbidity has been associated with decreased physical functioning [10, 11], lower quality of life [11], higher mortality [12], and increased healthcare service use and cost [13, 14]. It is now regarded as one of the largest global healthcare challenges of the twenty-first century [6, 7, 15].

This paper is concerned with the impact of multimorbidity on healthcare utilization, a topic for which detailed knowledge is incomplete [16]. Lehnert et al.’s systematic review [13] found that the majority of the 35 included (observational) studies showed a positive association between multimorbidity and service use/cost in older adults. This review and studies published after it note that while multimorbidity and healthcare service use are strongly related, the evidence has been inconsistent on how other socio-demographic factors known to be independently related to multimorbidity and healthcare service use impact the relationship between the two [13, 17].

Studies, most published since the Lehnert et al. review, have identified a range of factors affecting healthcare service use in older adults with multimorbidity, including age and sex [17–21], socio-economic status [17], eligibility for free medical care [18], living alone [22], and impaired activities of daily living [19]. However, the results for these factors have been inconsistent. Van den Bussche et al. [19] found that service use was related to multimorbidity, but no differences in this relationship were seen across sex or age groups. Fortin et al. [23] found sex differences where multimorbidity was higher in women than men yet men had higher healthcare use. Results similar to Fortin et al.’s have been reported by others [17, 19]. Inconsistent age/sex interactions have been seen, with one study showing that increasing age was associated with increasing physician consultations in men and decreasing consultations in women [17], and another showing a larger age effect on consultations in women versus men [18]. Some studies have found lower socio-economic status was associated with lower healthcare service use [17], and others have found no association [24]. This heterogeneity of study findings can reflect different levels of multimorbidity [25], inaccurate or incomplete correlates data [17], and geographical differences in healthcare systems and care delivery models [15, 26].

Many studies to date have treated socio-demographic factors as covariates that are adjusted for in regression analyses examining the relationship between multimorbidity and healthcare service use, or examined only a few socio-demographic factors. Adjusting for confounders in regression models does not explore potential effect modification, i.e., whether the relationship between multimorbidity and healthcare service use is consistent across levels of the socio-demographic factors. In many practice settings, resources to implement interventions are limited, thus it may not be possible to treat an entire population. Information on moderator and interaction effects can help to identify groups of individuals who would or would not benefit, or who would benefit to the greatest extent, from an intervention. Even in settings where resources are not limited, some individuals may benefit and others not from an intervention, in which case resources should target those that benefit and refrain from treating those that do not. Also, it may not be possible to intervene directly on the primary exposure of interest, such as multimorbidity. In such cases, intervention design can turn to the modifiable risk factors that eliminate much or some of the effect of the primary exposure on the outcome (healthcare service use in this study). Finally, studies that examine only a few socio-demographic factors may be missing key confounders. Therefore, it is important to consider a broad range of factors to better understand the role of multimorbidity relative to these other factors in shaping healthcare service use.

The purpose of this study was to examine the relationship between multimorbidity and acute care service use (i.e., hospital and emergency department visits) in older adults in Ontario, Canada, and how socio-demographic factors impact this relationship. We focused on acute care services because these are among the most expensive health care services, thus of great interest to policy and decision makers. We used a large-scale population-based survey linked to administrative data to explore the associations among multimorbidity, acute care service use, and a comprehensive range of socio-demographic factors. The findings can inform future explanatory theories that deepen our understanding of multimorbidity and its impacts.

## Methods

### Study design and setting

This is a retrospective cohort study in which we used data from four cycles of the Canadian Community Health Survey (CCHS) linked with health administrative data from Ontario, Canada’s largest province. Ontario has a population of approximately 14 million residents with the vast majority receiving provincial health insurance coverage for acute care services. Health administrative databases were used to obtain the one-year health service use from the index date of each of the four CCHS cycles. Since the distribution of health service use and other variables were similar over the time spanning the four CCHS cycles, we pooled the results for the CCHS cycles.

### Data sources

The CCHS is a national cross-sectional survey that collects information related to health status, health care utilization, and health determinants for the Canadian population. CCHS cycles 2005–2006 [27], 2007–2008 [28], 2009–2010 [29], and 2011–2112 [30] were chosen to maximize sample size and ensure consistency in the framing of questions relating to CCHS items used in this study. The four CCHS cycles were administered in participants’ homes using computer-assisted personal interviewing and participants in Ontario were asked if they would consent to have their CCHS data linked to provincial administrative data holdings. The index date for linkage was the participant’s CCHS interview date. Administrative databases used in the study were the: Registered Persons Database (demographics); Ontario Health Insurance Plan (OHIP) (physician visits); Discharge Abstract Database (inpatient hospitalizations); National Ambulatory Care Reporting System (emergency department other ambulatory contacts); Same Day Surgery (same-day surgeries, procedures); and Ontario Drug Benefit (outpatient prescription claims). Two additional data sources were accessed for specific diagnostic information on chronic conditions: the Ontario Mental Health Reporting System and the Ontario Cancer Registry. More information on theses databases is provided in Additional File 1. All data are held at ICES, where they were linked using encoded identifiers and analyzed. ICES is an independent, non-profit research institute funded by an annual grant from the Ontario Ministry of Health and Long-Term Care. As a prescribed entity under Ontario’s privacy legislation, ICES is authorized to collect and use health care data for the purposes of health system analysis, evaluation and decision support. Secure access to these data is governed by policies and procedures that are approved by the Information and Privacy Commissioner of Ontario. The study received approval from the Hamilton Integrated Research Ethics Board at McMaster University (certificate #13–590) and renewed yearly as required.

### Study sample

**Fig. 1:**
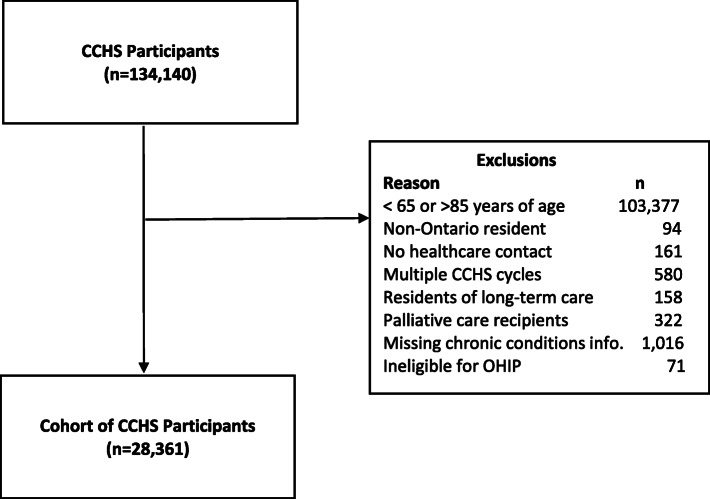
Study cohort of Canadian Community Health Survey participants (Ontario) who consented to administrative data linkage. Legend: CCHS - Canadian Community Health Survey, OHIP – Ontario Health Insurance Plan

We included Ontario CCHS participants who responded to any of the included CCHS cycles and who agreed to have their data linked to the health administrative data. We excluded those who were under 65 or over 85 years of age (*n*=103,377) because those under 65 to have health service use substantially different from older adults [31] and there was only a small number of CCHS participants over age 85. We excluded people who could not be identified as Ontario residents (*n*=94), who did not have health system contact within the 5 years prior to their survey date (*n*=161), who resided in long-term care (*n*=158) or received hospice or palliative care services (*n*=322), who participated in more than one CCHS cycle (we chose only the first cycle to avoid duplicate participants in the pooled data, *n*=580), who did not report their chronic disease status (*n*=1016) and who were ineligible for OHIP coverage at index (*n*=71). The final sample included 28,361 individuals (see Fig. 1).

### Chronic conditions and multimorbidity

We identified 12 chronic conditions: Alzheimer’s diseases/dementia, anxiety/depression, arthritis, cancer, asthma, chronic obstructive pulmonary disease (COPD), diabetes, heart disease, hypertension, inflammatory bowel disease, stomach or intestinal ulcers, and stroke. These conditions were chosen because they could be identified in the administrative data, are prevalent in older adults, and are frequently reported in the literature on multiple chronic conditions [7, 32, 33]. Chronic conditions were identified as either entry into a disease-specific database created at ICES or using an algorithm that searched for specific diagnostic codes and/or outpatient prescription claims within the 5 years prior to baseline. More details about each diagnostic definition can be found in Additional File 2.

Multimorbidity was operationalized as a count of individual chronic conditions (0, 1, 2, 3, 4, or 5 or more).

### Acute care service use

The outcome in this study was acute care service use, hospital admissions and emergency department visits that occurred in the 12 months after the CCHS interview date. The analyses included two measures for each service: a dichotomous variable (any visit/admission over one year) and a count variable (number of visits/admissions over one year).

### Socio-demographic & Health Status Variables

The socio-demographic and health status variables selected for the study were determined by those available from the CCHS, guided by Anderson and Newman’s Behavioural Model of Health Care Utilization which identifies the following 3 determinants of health service use: need, enabling, and predisposing factors [34, 35]. Need is determined by a person’s perceived need for health services, which in turn is a function of self-perceived health, activities of daily living (ADLs) or restricted activity, self-reported symptoms, quality of life, etc. Enabling factors include a person’s income, health insurance status, and access to regular care. Predisposing factors include demographic variables, attitudes, and beliefs. Anderson and Newman’s model has been primarily used for explaining health care utilization in the general population, with strong evidence of a socio-economic gradient in both developed and developing countries [36–40]. Our goal in using the Anderson and Newman model was to ensure that we were comprehensive in capturing the main determinants that have been hypothesized to shape health service use. Predisposing variables in our study included sex, age, marital status and living arrangement; enabling factors included education, household income, and rurality; and needs-based variables were captured by the need for any assistance with daily activities, and two health-status variables (self-perceived physical and mental health). Additional File 3 provides further details on how these variables were defined and operationalized in the study.

Multiple imputation using a discriminant function method [41] was employed to address missing data, which only occurred for certain CCHS variables. All CCHS data and chronic conditions and a flag indicating death during a five-year prospective observation period from administrative data were used to impute missing data. Additional file 3 shows the amount of missing data for each variable, with household income have the highest level of missing data (7.56%), followed by education (3.22%) and self-reported mental health (2.25%).

### Statistical analysis

Our main interest was the role of socio-demographic and health status factors in moderating the association between multimorbidity and acute care service use, in other words, the interaction of these variables with multimorbidity to influence service use. We began by conducting bivariate analyses exploring the association between multimorbidity and acute care service use stratified by each socio-demographic and health status variable. Logistic regression was employed in the bivariate analysis, modelling any vs. no acute care service over one year. All odds ratios (ORs) used a reference of 0 chronic conditions for one of the subgroups, e.g., the OR for any hospitalization during the past year for females and males with 1 chronic condition represent the odds in those with 1 chronic condition compared to females with no chronic conditions. Analyses showing differences in the multimorbidity-service use relationship across strata, were followed up with more detailed stratified analyses to determine whether the patterns were similar across demographic subgroups. For example, a difference in the relationship between multimorbidity and service use between males and females was followed with further stratified analyses (e.g., age and sex) to determine if the sex difference was similar across age groups. Given the large number of socio-demographic and health status variables in the study, it was not feasible to explore the interactions of multimorbidity with all variables. Therefore, we examined the patterns in the stratified analyses, and identified a subset of variables for which the relationship between multimorbidity and acute care service use showed evidence of significant variability across subgroups. Because of the significant role of age and sex in healthcare analyses, the regression models that explored interaction effects were run separately for each age/sex stratum (i.e., males 65–74 years, males 75–84 years, females 65–74 years, females 75–84 years). We also performed multivariable regressions, which included multimorbidity and all the socio-demographic and health status variables, to adjust for these factors in exploring the association between multimorbidity and acute care service use. Logistic regression was used for the dichotomous dependent variable (any versus no health service use) and Poisson regression was used to analyze the number of health service encounters. Interactions between multimorbidity and the covariates were explored in the regressions. Regressions did not include the CCHS cycle as a variable because early analyses showed no evidence of changes in sociodemographic or service use variables over time.

SAS version 9.4 was used for all statistical analyses, and the level of significance used throughout the study was alpha=0.05.

## Results

### Socio-demographic, health status and acute care service use

Of the 28,361 members of the study sample, 60% were between the ages of 65 and 74 years, 57% were female, 72% were non-immigrant, and over 75% lived in an urban area and had annual household incomes below $80,000. While 72% perceived their mental health to be very good or excellent, fewer (46%) perceived their physical health to be so and 44% reported having 3+ chronic conditions. The majority (80%) did not need help with basic daily tasks, such as meal preparation, routine household errands, personal care etc. During the previous year, 7% had at least one emergency department visit and 12% had at least one acute care episode (see Table 1).

**Table 1:** Prevalence of selected socio-demographic and health status characteristics, multimorbidity and acute care service use

Characteristic	Category	Frequency (***n***)	Percentage (%)
Age	65–74	16,979	59.87
	75–84	11,382	40.13
Sex	Male	12,093	42.64
	Female	16,268	57.36
Immigrant Status	Immigrant	7736	27.28
	Non-Immigrant	20,625	72.72
Education	Post Secondary Degree	12,661	44.64
	Secondary School Degree	6139	21.65
	No Diploma	9561	33.71
Household Income	Under $30,000	10,382	36.61
	$30,000 to $79,999	13,723	48.39
	$80,000 or more	4256	15.01
Living Arrangement	Living with others	16,583	58.47
	Living Alone	11,778	41.53
Geography	Urban	21,402	75.46
	Rural	6959	24.54
Instrumental Activities of Daily Living (IADLs)	Does not need help with basic tasks	22,732	80.15
	Needs help with basic tasks	5629	19.85
Self perceived physical health	Excellent/Very Good	12,937	45.62
	Good	9027	31.83
	Fair or Poor	6397	22.56
Self perceived Mental Health	Excellent/Very Good	20,369	71.82
	Good	6490	22.88
	Fair or Poor	1502	5.3
Chronic Conditions	0	2457	8.7
	1	5786	20.4
	2	7694	27.1
	3+	12,424	43.80
Emergency Department Visits	Any visit (past year)	1895	6.7
	Mean (standard deviation)	1.74 (1.76)	
Acute Care Episode (Hospital or Emergency Department Visit)	Any episode (past year)	3515	12.4
	Mean (standard deviation)	1.34 (0.73)	

### Stratified analyses: relationship between acute care service use and multimorbidity

**Fig. 2:**
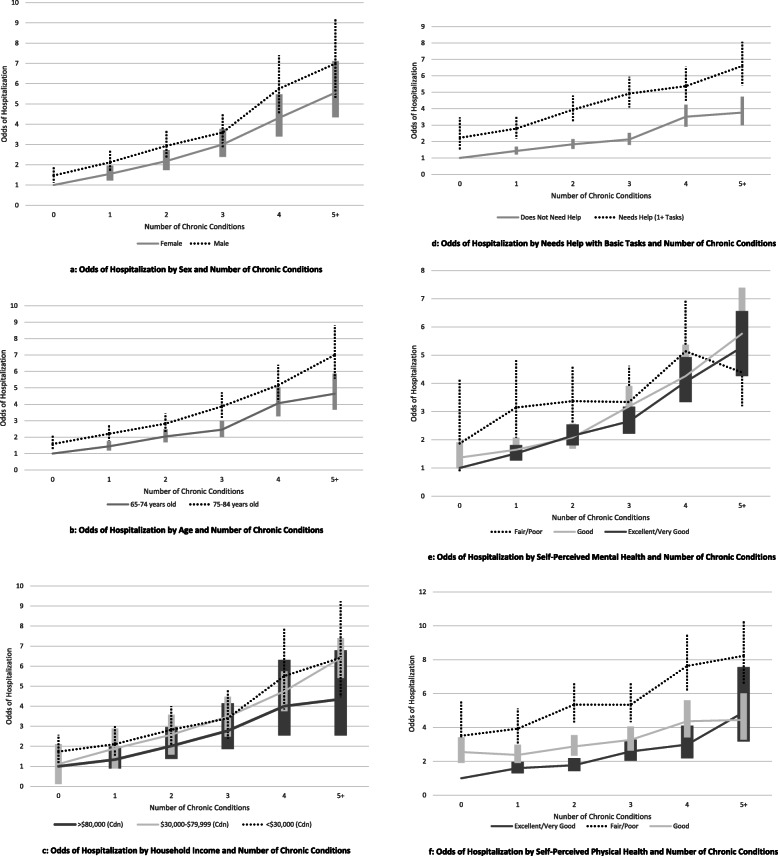
**a-f. a**: Odds of Hospitalization by Sex and Number of Chronic Conditions. **b**: Odds of Hospitalization by Age and Number of Chronic Conditions. **c**: Odds of Hospitalization by Household Income and Number of Chronic Conditions. Odds of Hospitalization by Needs Help with Basic Tasks and Number of Chronic Conditions. **e**: Odds of Hospitalization by Self-Perceived Mental Health and Number of Chronic Conditions. **f**: Odds of Hospitalization by Self-Perceived Physical Health and Number of Chronic Conditions

Given the large number of stratification variables, the main body of the paper provides the figures where key relationships were seen for any hospital use, with the remaining stratified figures for hospital use provided in Additional File 4 and all figures for any emergency department use provided in Additional File 5. Fig. 2a-f show the odds ratios (ORs) for any hospital use within 1 year of CCHS index date by level of multimorbidity (number of chronic conditions), stratified by *selected* socio-demographic and health status variables, i.e., sex, age, household income, needs help, self-perceived mental health and self-perceived physical health.

There was little evidence of effect modification across the range of socio-demographic and health status variables. Hospitalizations and emergency department visits consistently increased with the level of multimorbidity and stratified analyses revealed further patterns independent of the level of multimorbidity. The ORs for both services were higher at all levels of multimorbidity for: men (vs women) (Fig. 2a, Fig. 5 a – Additional File 5), older age groups (age 75–84 vs 65–74) (Fig. 2c, Fig. 5 b – Additional File 5), those with lower annual household income (below $30,000 vs above) (Fig. 2e, Fig. 5 c – Additional File 5), those that needed help with daily tasks (Fig. 2d, Fig. 5 d – Additional File 5), those with lower perceived mental and physical health (Fig. 2e & f, Figs. 5 e & f – Additional File 5), and those with less education (Fig. 4 d - Additional File 4, Fig. 5 j – Additional File 5). Different OR patterns for the two services were seen for rurality and immigrant status, which did not appear to impact hospitalizations (Figs. 4 b and c – Additional File 4) yet impacted emergency department use (higher in rural vs urban residents and non-immigrants vs immigrants) (Figs. 5 h & i – Additional File 5). The ORs for both services did not differ substantially by marital status (Fig. 4 a – Additional File 4, Fig. 5 g – Additional File 5) or living arrangement (Fig. 4 e – Additional File 4, Fig. 5 k – Additional File 5).

### Interaction results

We examined the interactions between the following three variables and multimorbidity based on an assessment of the patterns seen in the stratified analyses: household income, needing help with basic tasks, and self-perceived mental health. Table 2 provides the results from this analysis for any hospital use, and Additional File 6 provides the results for any emergency department visit. Twelve regression models were run (three variables x four age/sex strata) for each type of acute care use, with very few models showing significant interactions. Only two of the twelve models showed a significant interaction effect for any hospital use (*p*=0.0009, self-perceived mental health in males 75–84 years; *p*=0.0001, needing help with basic tasks in females 75–84 years) and only one model showed a significant interaction effect for any emergency department use (*p*=0.04, household income in females 65–74).

**Table 2:** Interaction Effects Analysis for Hospitalizations – Multiple Imputation Results

Sex, Age Group	MM^**a**^ Group	Correlate Group	Overall N (% with 1+ Hosp)	OR (95% CI)^**b**^	Inter.^**c**^ (***p***-value)
**Interaction #1: MM x Income**					
Female, 65–74	0–1	>$80 k	640 (3)	Ref	0.11
		**$30 k-$79.9 k**	**1950 (7)**	**2.25 (1.39, 3.63)**	
		**<$30 k**	**1286 (6)**	**1.95 (1.18, 3.22)**	
	2–3	>$80 k	507 (8)	Ref	
		$30 k-$79.9 k	1955 (10)	1.22 (0.86, 1.74)	
		<$30 k	1672 (11)	1.36 (0.96, 1.94)	
	4+	>$80 k	108 (15)	Ref	
		$30 k-$79.9 k	563 (16)	1.08 (0.60, 1.92)	
		<$30 k	749 (20)	1.42 (0.81, 2.48)	
Female, 75–84	0–1	>$80 k	210 (9)	Ref	0.20
		$30 k-$79.9 k	937 (7)	0.82 (0.47, 1.41)	
		<$30 k	962 (10)	1.20 (0.71, 2.03)	
	2–3	**>$80 k**	**285 (9)**	**Ref**	
		**$30 k-$79.9 k**	**1421 (15)**	**1.65 (1.08. 2.51)**	
		<$30 k	1719 (15)	1.63 (1.08, 2.48)	
	4+	>$80 k	102 (20)	Ref	
		$30 k-$79.9 k	429 (23)	1.21 (0.71, 2.08)	
		<$30 k	773 (24)	1.32 (0.79, 2.21)	
Male, 65–74	0–1	>$80 k	911 (7)	Ref	0.52
		$30 k-$79.9 k	1805 (7)	1.10 (0.80, 1.51)	
		**<$30 k**	**804 (10)**	**1.61 (1.14, 2.28)**	
	2–3	>$80 k	686 (11)	Ref	
		$30 k-$79.9 k	1752 (13)	1.26 (0.95, 1.66)	
		<$30 k	798 (14)	1.36 (0.99, 1.87)	
	4+	>$80 k	107 (16)	Ref	
		$30 k-$79.9 k	407 (23)	1.57 (0.89, 2.77)	
		<$30 k	279 (25)	1.77 (0.99, 3.19)	
Male, 75–84	0–1	>$80 k	253 (8)	Ref	0.65
		$30 k-$79.9 k	866 (12)	1.47 (0.90, 2.41)	
		**<$30 k**	**483 (16)**	**2.06 (1.24, 3.43)**	
	2–3	>$80 k	350 (15)	Ref	
		$30 k-$79.9 k	1238 (17)	1.15 (0.83, 1.59)	
		<$30 k	616 (20)	1.34 (0.94, 1.90)	
	4+	>$80 k	97 (21)	Ref	
		$30 k-$79.9 k	400 (28)	1.46 (0.85, 2.50)	
		<$30 k	241 (30)	1.64 (0.93, 2.88)	
**Interaction #2: MM x Self-Perceived Mental Health**					
Female, 65–74	0–1	Excellent	3192 (6)	Ref	0.97
		Good	627 (7)	1.15 (0.81, 1.63)	
		Fair/Poor	57 (< 11)^d^	1.24 (0.44, 3.47)	
	2–3	Excellent	3040 (9)	Ref	
		Good	900 (12)	1.27 (1.00, 1.62)	
		Fair/Poor	194 (12)	1.31 (0.83, 2.06)	
	4+	Excellent	828 (17)	Ref	
		Good	415 (19)	1.12 (0.82, 1.52)	
		Fair/Poor	177 (20)	1.26 (0.83, 1.89)	
Female, 75–84	0–1	Excellent	1633 (8)	Ref	0.53
		Good	435 (9)	1.12 (0.77, 1.63)	
		Fair/Poor	41 (17)	2.28 (0.99, 5.25)	
	2–3	Excellent	2404 (14)	Ref	
		Good	864 (15)	1.05 (0.84, 1.31)	
		Fair/Poor	257 (16)	1.17 (0.75, 1.82)	
	4+	Excellent	740 (23)	Ref	
		Good	383 (24)	1.04 (0.78, 1.40)	
		Fair/Poor	181 (23)	0.99 (0.68, 1.47)	
Male, 65–74	0–1	Excellent	2792 (8)	Ref	0.65
		Good	643 (8)	1.06 (0.77, 1.46)	
		Fair/Poor	85 (13)	1.83 (0.96, 3.50)	
	2–3	Excellent	2257 (13)	Ref	
		Good	805 (12)	0.96 (0.75, 1.22)	
		Fair/Poor	174 (13)	1.05 (0.67, 1.66)	
	4+	Excellent	444 (21)	Ref	
		Good	242 (23)	1.10 (0.75, 1.60)	
		Fair/Poor	107 (29)	1.52 (0.94, 2.44)	
Male, 75–84	0–1	Excellent	1173 (12)	Ref	**0.0009**
		Good	371 (13)	1.19 (0.84, 1.68)	
		**Fair/Poor**	**58 (22)**	**2.20 (1.16, 4.19)**	
	2–3	Excellent	1459 (17)	Ref	
		Good	597 (17)	1.03 (0.80, 1.33)	
		**Fair/Poor**	**148 (30)**	**2.11 (1.45, 3.09)**	
	4+	Excellent	407 (29)	Ref	
		Good	208 (30)	1.07 (0.74, 1.54)	
		Fair/Poor	123 (20)	0.61 (0.37, 1.00)	
**Interaction # 3: MM x Instrumental Activities of Daily Living**					
Female, 65–74	0–1	Does not need help	3575 (5)	Ref	0.49
		**Needs help**	**301 (12)**	**2.48 (1.71, 3.61)**	
	2–3	Does not need help	3372 (8)	Ref	
		**Needs help**	**762 (17)**	**2.27 (1.81, 2.84)**	
	4+	Does not need help	833 (14)	Ref	
		**Needs help**	**587 (24)**	**1.92 (1.46, 2.52)**	
Female, 75–84	0–1	Does not need help	1750 (8)	Ref	**0.0001**
		**Needs help**	**359 (12)**	**1.64 (1.14, 2.34)**	
	2–3	Does not need help	2235 (10)	Ref	
		**Needs help**	**1190 (22)**	**2.38 (1.96, 2.89)**	
	4+	Does not need help	526 (22)	Ref	
		Needs help	778 (25)	1.18 (0.91, 1.54)	
Male, 65–74	0–1	Does not need help	3373 (7)	Ref	0.89
		**Needs help**	**147 (16)**	**2.33 (1.46, 3.70)**	
	2–3	Does not need help	2895 (11)	Ref	
		**Needs help**	**341 (22)**	**2.18 (1.64, 2.88)**	
	4+	Does not need help	575 (18)	Ref	
		**Needs help**	**218 (35)**	**2.42 (1.71, 3.44)**	
Male, 75–84	0–1	Does not need help	1430 (11)	Ref	0.25
		**Needs help**	**172 (20)**	**1.98 (1.31, 2.96)**	
	2–3	Does not need help	1752 (15)	Ref	
		**Needs help**	**452 (29)**	**2.36 (1.85, 3.01)**	
	4+	Does not need help	416 (23)	Ref	
		**Needs help**	**322 (33)**	**1.68 (1.21, 2.33)**	

^a^ *MM* multimorbidity

^b^ OR (95% CI) = Odds Ratio of 1+ Hospitalization (95% Confidence Interval)

^c^ *Inter* Interaction

^d^ (%) with 1+ hospitalizations rounded upward to avoid reporting of small cell size

### Multivariable regression results

In an unadjusted regression model, the odds of any hospitalization for those with 2–3 and 4+ chronic conditions compared to 0–1 chronic conditions was 1.74 and 3.30, respectively. Table 3 shows the results for the adjusted multiple regression models for any hospitalization and the number of hospitalizations, with all correlates simultaneously included in the model. The ORs for multimorbidity are reduced once other correlates are included in the model (to 1.31 for 2–3 chronic conditions and 1.84 for 4+), but remain statistically significant in both models. The results were similar for both outcomes (any hospitalization, number of hospitalizations), with higher odds (or incidence) of hospitalization seen in those with higher levels of multimorbidity, in older respondents, lower household income, lower perceived physical health, and in those that were male, non-immigrant, needed help with basic tasks or lived alone. Odds of hospitalization were *lower* in those with lower perceived mental health. Education and residency (urban/rural) were not associated with either hospitalization outcome.

**Table 3:** Odds Ratios and Incidence Rates of Hospitalization, Multiple Imputation Results

Variable	OR [95% CI]^**a**^	IR [95% CI]^**a**^
**Chronic Conditions**		
0–1	–	–
2–3	**1.31 [1.20, 1.44]**	**1.35 [1.24, 1.47]**
4+	**1.84 [1.64, 2.06]**	**1.80 [1.61, 2.00]**
**Age**		
65–74	–	–
75–84	**1.32 [1.22, 1.42]**	**1.29 [1.20, 1.39]**
**Sex**		
Female	–	–
Male	**1.45 [1.34, 1.57]**	**1.43 [1.32, 1.54]**
**Immigrant Status**		
Immigrant	–	–
Non-Immigrant	**1.16 [1.06, 1.26]**	**1.14 [1.05, 1.24]**
**Education**		
Post-secondary degree	–	–
Secondary school degree	0.95 [0.86, 1.05]	0.98 [0.89, 1.08]
No diploma	1.08 [0.99, 1.18]	**1.11 [1.02, 1.21]**
**Household Income**		
Over $80,000	–	–
$30,000 to $79,999	**1.22 [1.07, 1.38]**	**1.30 [1.15, 1.47]**
Under $30,000	**1.22 [1.06, 1.40]**	**1.28 [1.12, 1.47]**
**Living Arrangement**		
Living with others	–	–
Living alone	**1.15 [1.06, 1.25]**	**1.12 [1.04, 1.22]**
**Geography**		
Urban	–	–
Rural	1.04 [0.96, 1.14]	1.05 [0.96, 1.13]
**Instrumental Activities of Daily Living (IADLs)**		
Does not need help with basic tasks	–	–
Needs help with basic tasks	**1.68 [1.54, 1.84]**	**1.58 [1.45, 1.72]**
**Self-Perceived Physical Health**		
Excellent/Very Good	–	–
Good	**1.50 [1.36, 1.65]**	**2.30 [2.08, 2.55]**
Fair or Poor	**2.29 [2.05, 2.54]**	**1.51 [1.37, 1.65]**
**Self-Perceived Mental Health**		
Excellent	–	–
Good	**0.88 [0.81, 0.97]**	**0.90 [0.83, 0.98]**
Fair or Poor	**0.79 [0.68, 0.93]**	**0.84 [0.73, 0.97]**

^a^ OR [95% CI] = Odds Ratio [95% Confidence Interval], IR [95%CI] = Incidence Rate [95% Confidence Interval]

In the unadjusted regression model, the odds of any emergency department visit for those with 2–3 and 4+ chronic conditions compared to 0–1 chronic conditions were 1.53 and 2.84, respectively. Additional File 7 shows the results of the adjusted multiple regression for any ED visit and the number of ED visits. The ORs for multimorbidity are reduced once other correlates are included in the model (to 1.21 for 2–3 chronic conditions and 1.75 for 4+), but remain statistically significant in both models. The results for both outcomes (any emergency department use, number of emergency department visits) were similar and largely consistent with those discussed above for hospitalizations.

### Age and sex differences in service use

**Fig. 3:**
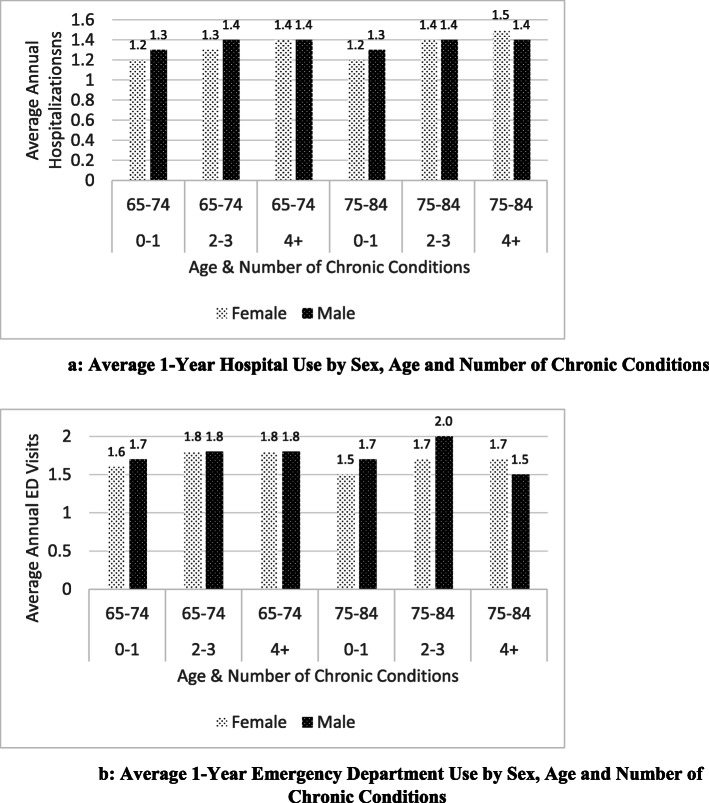
**a** and **b**: **a**: Average 1-Year Hospital Use by Sex, Age and Number of Chronic Conditions. **b**: Average 1-Year Emergency Department Use by Sex, Age and Number of Chronic Conditions

We explored service use differences by age and sex, since some studies report age/sex influences on health care use and/or cost. Figure 3a and b compare males and females respectively on the average hospitalization and ED use (among those with at least one ED visit or hospitalization) across age/multimorbidity strata. These figures do not suggest major sex differences across the age/multimorbidity strata. The largest difference between males and females occurs in the highest multimorbidity/age category (4+, 75–84) for ED visits, where females remain the same but males decline in use from an average of 2.0 to 1.5 visits compared to the lower multimorbidity category (2-3, 75–84).

## Discussion

### Summary of main findings

This study is one of the few to examine the influence of a comprehensive range of socio-demographic factors and health status factors on the relationship between multimorbidity and acute care service use. Our main interest was the role of these factors in moderating the association between multimorbidity and acute care service use. We did not observe strong evidence of moderator or interaction effects across this comprehensive range of factors. While most of the factors influenced acute care service use independent of multimorbidity, they did not change its fundamental relationship with service use - i.e., increased multimorbidity was consistently associated with increased service use for all subgroups.

### Comparison with existing literature

The vast majority of studies to date have examined the independent effects of selected socio-demographic and health status (e.g., multimorbidity) factors on health service use. We are not aware of many studies examining factors moderating the relationship between multimorbidity and service use, or interacting with multimorbidity in shaping service use, with the exception of those looking at age and sex. Overall, we did not find a significant age/sex interaction influencing acute care service use, consistent with other studies, including the study by van den Bussche et al. [19] looking at ambulatory care services and Hessel et al. [24] who studied general practitioner and specialist services. The drop we observed in ED visits for males in the highest multimorbidity/age category (4+, 75–84) compared to the lower multimorbidity/age category (2–3, 75–84) is unusual. While Librero et al. [42] reported a similar finding, the review by Lehnert et al. [13] noted that the Librero et al. [42] study was unusual, with all other studies showing a positive association between number of chronic conditions and ED visits. Further research may be help to understand our results (e.g., men in the highest multimorbidity category may spend more time in the hospital compared to the ED, women in the older age groups excluded from this study may show this same pattern).

The effects we observed between socio-demographic and health status factors and acute care service use independent of multimorbidity are consistent with much of the existing literature. The sex differences we observed were not seen in Canadian studies focused on ED use in older adults [43, 44], yet other studies show that older men dominate resource-intensive health care [12, 45–47] perhaps due to men having conditions that require these services [46] or seeking health care only when illness is acute/serious [48]. We observed higher odds of acute care service use in older adults (age 75–84 vs 65–74), for which ample evidence exists [46], including the independent (of multimorbidity) age effects seen in Payne et al.’s [49] large general population study and studies of various disease cohorts [50, 51]. Our finding that lower household income and education were associated with higher odds of acute care service use is consistent with another Canadian study [7] and others using a range of individual and/or regional socioeconomic status measures [17, 18, 52]. We observed that non-immigrants had higher use of acute care services compared to immigrants, consistent with Roberge et al.’s Canadian study on ED use [53] and thought to reflect the better health of new immigrants or language/knowledge-related access barriers [54]. We found higher ED use (not hospitalizations) among rural versus urban residents, which has been reported by other Canadian studies [53, 55] and attributed to access barriers or a perceived need for specialized services [56, 57] as well as the tendency of rural physicians to practice in EDs and hospitals and thus encourage patients to use these services [58, 59]. Our study found that low/poor self-perceived health and functionality were linked to higher acute care service use, consistent with other studies [60–63] and research showing that adding functional status to administrative-based chronic illness data improves the ability to identify high risk/high-cost system users [60, 64]. We found that lower perceived self-reported mental health was associated with lower hospital use (not ED use), unlike studies that show that mental health disorders are a key driver of higher ED use [65, 66]. However, we used self-reported mental health, which differs from objective (diagnostic-based) measures of mental illness [67–69], which may explain study differences. We also found that living alone was associated with higher hospitalizations (not ED use) as seen in some studies [70, 71] and not others [72], which is broadly consistent with a systematic review linking weaker social relationships with increased hospitalizations [73], associations seen between social isolation/loneliness and poor health [74, 75] and findings that social isolation/loneliness predicts ED use [66, 76–80].

### Implications of study findings for research, Policy & Practice

While our data showed that multimorbidity impacts on acute care service use similarly across most socio-demographic and health variables, it also shows that these variables have their own direct effect on acute care service use and should be considered in intervention development, resource targeting, and future research. The message appears to be - multimorbidity impacts acute care service use consistently and similarly across many population subgroups.

Our results suggest that we cannot ignore population subgroups where there is consistent evidence pointing to a higher risk of acute care service use, or where there are inconsistent findings that require further research. The multiple regression results from this study show that a number of factors are linked to higher acute care service use, independent of multimorbidity. Age is a strong predictor of health service use across many studies, thus targeting resources and designing interventions to support older age groups is warranted to enhance access to community-based supports and assist them in managing conditions other than chronic conditions that may explain higher acute care use (e.g., geriatric syndromes such as frailty, incontinence, pain). Physical health and functionality, which are linked to age and multimorbidity but also independent of these, are strong predictors of service use in many studies. Taking a broader view of physical health that includes functional limitations and self-reported health status measures can be a source of potent predictors of service needs/use. Including these measures in standard clinical assessments can be done, which could then inform resource targeting and intervention design. Consistent evidence links low socioeconomic status with higher acute care service use, even in Canada where there is a national system of health and social services. This socioeconomic gradient is independent of multimorbidity, which itself is more prevalent in socioeconomically-deprived populations [81]. Targeting resources and interventions at low socio-economic groups can help to remove barriers to accessing healthcare services [82] and avoid the cascading effects resulting from developing poorer health outcomes that require more expensive healthcare services [7]. Some factors require further research in order to determine if an intervention is needed and what it should target. For example, immigrant’s use of acute care services is less than non-immigrant’s use, but why - does this reflect health differences or access barriers? Rural residents use more acute care services, but why – does this reflect the need for specialized or immediate services, accessibility, or family physician directives? Studies from Canada [55, 83] and the U.S. [84] suggest that high users of primary care are also high ED users, so access may not be the main driver in the acute care service use of rural residents. Mental health and living status may either increase, decrease or not affect acute care service use. These differences need to be better understood, and would likely benefit from the use of better and more consistent construct measures.

Finally, this study should be seen as part of an ongoing research agenda aimed at untangling the complex relationships between multimorbidity, health service use, and various socio-demographic and health status variables. Important next steps include longitudinal research to explore the potential mediating role that these variables may play in the relationship between multimorbidity and health service use.

### Strengths and limitations

A major strength of this study was the use of a large sample of Canadian older adults and administrative data with robust and well-reported measures of chronic conditions and accurate capture of health service use. By linking to the CCHS, we were able to incorporate a comprehensive range of sociodemographic variables that are typically not available when using administrative data alone. Other strengths include minimal missing data, and modelling acute care service outcomes two different ways (any service and number of services) showing that this methodological choice did not significantly impact the findings from the multiple regression.

We also acknowledge the key limitations of our study. Our measure of multimorbidity included 12 chronic conditions. These conditions represent the most prevalent chronic conditions in Canadian older adults [85] and those often used in multimorbidity research [86]. However, there are conditions other than those captured in this study that may influence healthcare service use. Also, our measure of multimorbidity - the number of chronic conditions - does not capture differences in the types/clusters/severity of the conditions, which can also impact healthcare service use. Essentially, we still lack a consistent definition or framework for thinking about multimorbidity, which impacts research and the comparability of studies [14]. Our study also uses data from a large national survey (the CCHS), which has limitations including potential biases related to self-report data (e.g., response bias, recall bias) and module changes that limit comparing across or pooling CCHS cycles. Our study focused on acute care (hospital, emergency department) services; it would be useful to examine primary care and other service use for a more comprehensive understanding of impacts. We explored specific interactions between multimorbidity and the covariates, which was necessarily selective due to the large number of covariates in the study. Although few interaction terms were significant in the regressions, it is possible that some relevant interactions were missed.

## Conclusion

This study examined the relationship between multimorbidity and acute care service use and how various socio-demographic and health factors impact this relationship. The study did not reveal strong evidence of moderator or interaction effects across the range of factors, suggesting that multimorbidity interventions can be delivered broadly. Multimorbidity is consistently and similarly associated with acute care service use across many population subgroups, thus it is important to continue multimorbidity research to better understand its origins and impacts and target resources/programs to better manage it. This study also found that many factors influenced acute care service use independent of multimorbidity, pointing to modifiable risk factors that can be the focus of resource allocation and intervention design to reduce acute care service in those with multimorbidity. Ultimately, the study’s results suggest that optimizing acute care service use in older adults will require attention to both multimorbidity and social determinants, with programs that are multifactorial and integrated across the health and social service sectors.

## Supplementary Information

**Additional file 1.** Institute for Clinical Evaluative Science (ICES) Database Details.

**Additional file 2.** Diagnostic Definitions for 12 Chronic Conditions.

**Additional file 3.** Canadian Community Health Study Covariates, Operationalization, and Percent Missing.

**Additional file 4.** Remaining Stratified Figures for Odds of Hospitalization.

**Additional file 5.** Stratified Figures for Odds of Emergency Dept Visit.

**Additional file 6.** Interaction Effects Analysis for Emergency Department Visits – Multiple Imputation Results.

**Additional file 7.** Odds Ratios and Incidence Rates of Emergency Department Visits - Multiple Imputation Results.

## Data Availability

The data that support the findings of this study are available from the Institute for Clinical Evaluative Sciences (ICES), an independent, non-profit research institute funded by an annual grant from the Ontario Ministry of Health and Long-Term Care. However, restrictions apply to the availability of these data, which were used under license for the current study, and so are not publicly available. Those interested in acquiring these data must submit an application to ICES.

## Abbreviations

ADL: Activities of Daily Living
CCHS: Canadian Community Health Survey
COPD: Chronic Obstructive Pulmonary Disease
ED: Emergency Department
IADL: Instrumental Activities of Daily Living
ICES: Institute for Clinical Evaluative Sciences
LCL: Lower 95% Confidence Limit
OHIP: Ontario Health Insurance Plan
OR: Odds Ratio
UCL: Upper 95% Confidence Limit
