# Supplementary material for: Effect of socio-demographic and health factors on the association between multimorbidity and acute care service use: population-based survey linked to health administrative data
Source: BMC Health Serv Res. 2021 Jan 13;21:62. doi: 10.1186/s12913-020-06032-5 (PMC7805153; doi:10.1186/s12913-020-06032-5)
Supplement: Supplementary file 1 — Additional file 1. Institute for Clinical Evaluative Science (ICES) Database Details. [file 12913_2020_6032_MOESM1_ESM.docx]

**Additional File 1: Database Details**

| Discharge Abstract Database (DAD) | The DAD consists of data systematically extracted from inpatient hospital medical records by trained abstractors for all discharges from Ontario hospitals. The DAD includes demographic, clinical, and administrative data elements. The clinical data include diagnoses captured using International Classification of Disease codes (version 9 or 10, depending on year). The DAD is compiled by the Canadian Institute for Health Information (CIHI).  See: Juurlink et al. Canadian Institute for Health Information Discharge Abstract Database: A Validation Study. 2006. Toronto, Institute for Clinical Evaluative Sciences. |
| --- | --- |
| National Ambulatory Care Reporting System (NACRS) | The NACRS database consists of data collected during all ambulatory care encounters at hospitals in Ontario, including emergency department visits. Data elements include administrative information such as various date and time stamps and type of service, clinical information captured using ICD, 10^th^ revision diagnostic codes.  The Same Day Surgery (SDS) database is derived from NACRS.  See: Data Quality Documentation, National Ambulatory Care Reporting System – Current Year Information, 2015-2016 at https://www.cihi.ca/sites/default/files/document/nacrs-dataquality_2015-2016_en.pdf |
| Ontario Drug Benefit (ODB) program database | The ODB database contains claims for all prescriptions covered under the ODB program filled at outpatient pharmacies in Ontario. The ODB program provides prescription medication coverage for individual aged 65 and older and others meeting specific criteria.  See: Levy et al. Coding accuracy of administrative drug claims in the Ontario Drug Benefit database. Canadian Journal of Clinical Pharmacology 2003; 10(2): 67-71. |
| Ontario Health Insurance Plan (OHIP) claims history database | The OHIP claims database includes all claims to the program made by physicians providing care within Ontario. The majority of Ontario residents, with certain exceptions, receive coverage for physician services through the OHIP program. OHIP claims include those made through fee-for-service activities and “shadow” billings for those made through alternative funding arrangements. OHIP claims include a reason for the visit coded using a modified version of the ICD, 8^th^ revision.  See: Williams & Young. A summary of the quality of health care administrative databases in Canada. In Goel et al., editors. Patterns of Health Care in Ontario: The ICES Practice Atlas. 2^nd^ edition. Ottawa: Canadian Medical Association, 1996. 339-346. |
| OHIP Registered Persons Database (RPDB) | The RPDB consists of basic demographic information on all individuals with a valid OHIP insurance number. These data include age, sex, recent postal code, and data of death (where applicable). |
| Ontario Mental Health Reporting System (OMHRS) | The OMHRS captures data on all admissions to adult mental health beds in Ontario. Data is collected using the Resident Assessment Instrument – Mental Health (RAI-MH) clinical assessment tool which includes mental health diagnoses.  See: Ontario Mental Health Reporting System Metadata at <https://www.cihi.ca/en/ontario-mental-health-reporting-system-metadata> (last accessed on June 25, 2019) |
