# Supplementary material for: Effect of socio-demographic and health factors on the association between multimorbidity and acute care service use: population-based survey linked to health administrative data
Source: BMC Health Serv Res. 2021 Jan 13;21:62. doi: 10.1186/s12913-020-06032-5 (PMC7805153; doi:10.1186/s12913-020-06032-5)
Supplement: Supplementary file 2 — Additional file 2. Diagnostic Definitions for 12 Chronic Conditions. [file 12913_2020_6032_MOESM2_ESM.docx]

**Additional File 2: Diagnostic Definitions for 12 Chronic Conditions**

| Chronic Condition | Administrative Diagnostic Code |
| --- | --- |
| Alzheimer’s Disease/ Dementia | Any of the following in 5 years prior to index date:  ≥1 Hospitalization (DAD/SDS) with any of:  ICD-9: 290.0, 290.1, 290.3, 290.4, 290.8, 290.9, 294.1, 294.8, 294.9, 331.0, 331.1, 331.2, 797;  ICD-10: F00.0, F00.1, F00.2, F00.9, F01.0, F01.1, F01.2, F01.3, F01.8, F01.9, F02.0, F02.1, F02.2, F02.3, F02.4, F02.8, F03, F05.1, F06.5, F06.6, F06.8, F06.9, F09, G30.0, G30.1, G30.8, G30.9, G31.0, G31.1, R54;  OR  ≥1 ODB claim for cholinesterase inhibitors (donepezil, galantamine, or rivastigmine) DIN: 02232043, 02232044, 02269457, 02269465, 02244298, 02244299, 02244300, 02244302, 02266717, 02266725  OR  ≥3 physician visits (OHIP) at least 30 days apart in a two-year period with:  OHIP DXCODE: 290, 331, 797 |
| Anxiety and/or Depression | Any of the following in the 5 years prior to index date:  ≥1 Hospitalization with a diagnosis for depressive disorder, affective psychoses, neurotic depression or adjustment reaction: ICD-10-CA codes F31, F32, F33, F341, F38.0, F38.1, F41.2, F43.1, F43.2, F43.8, F53.0, F93.0 or with a diagnosis for an anxiety state, phobic disorders or obsessive-compulsive disorders: ICD-10-CA codes F40, F41.0, F41.1, F41.3, F41.8, F41.9, F42, F45.2  OR  ≥1 Hospitalization with a diagnosis for anxiety disorders: ICD-10-CA codes F32, F341, F40, F41, F42, F44, F45.0, F45.1, F45.2, F48, F68.0, or F99 AND one or more prescriptions for an antidepressant or mood stabilizer: ATC codes N05AN01, N05BA, N06A  OR  ≥1 physician visits with a diagnosis for depressive disorder or affective psychoses: ICD-9-CM codes 296, 311  OR  ≥1 physician visits with a diagnosis for anxiety disorders: ICD-9-CM code 300 AND one or more prescriptions for an antidepressant or mood stabilizer: ATC codes N05AN01, N05BA, N06A  OR  ≥3 physician visits with a diagnosis for anxiety disorders or adjustment reaction: ICD-9-CM code 300, 309  OMHRS: Section Q, item 1Mood/anxiety = yes if f or g = 1, 2, or 3 on discharge assessment  OR  Substance abuse/ addictions:  OMHRS Section Q, item 1 Mood/anxiety = yes if d= 1,2 or 3 |
| Arthritis | Any of the following in the 5 years prior to index date:  ≥1 Hospitalization (DAD, SDS) or physician visit (OHIP):  OHIP: 274, 446, 710, 711, 714, 715, 716, 718, 720, 727, 728, 729, 739  ICD-10: M05-M06, M15-M19, M07, M10, M11-M14, M30-36, M00-M03, M20-M25, M65-M79 |
| Asthma  [Cumulative registry from 1993 to 2016 using DAD, SDS, OHIP] | Cumulative ICES-created disease-specific database from 1993 to 2006 using DAD, SDS, and OHIP for:  ≥1 Hospitalization with a diagnosis of asthma  OR  ≥2 OHIP visits for asthma  ICD-9: 493  ICD-10: J45, J46 |
| Cancer | Inclusion in Ontario Cancer Registry (OCR) (from 1964 through index date) |
| Chronic Obstructive Pulmonary Disease | Cumulative ICES-created disease-specific database from 1996 to 2016 using DAD, SDS, and OHIP:  ≥1 Hospitalization with a diagnosis of COPD  OR  ≥2 OHIP visits for COPD  ICD-9: 491, 492, 496  ICD-10: J41, J42, J43, J44 |
| Diabetes | Cumulative ICES-created disease-specific database from 1991 to 2016 using DAD, SDS, OHIP, and ODB:  Ontario Diabetes Database, based on the following:  ≥1 Hospitalization (DAD,SDS)  ICD-9: 250  ICD-10: E10, E11, E13, E14  OR  From OHIP, 2 diagnostic codes within 1 year or 1 fee code:  Diagnostic code: 250  Fee code: Q040, K029, K045, K046  OR  One ODB diabetes mellitus drugs claim in a 1 year period |
| Heart Disease | Any of the following in the 5 years prior to index date:  Two physician billings within a one-year period with one of the billings by a specialist or a family physician in a hospital or emergency room setting) or a hospital discharge abstract.  ICD-10: I20-I25  CCP: 4802, 4803, 4809, 481  CCI: 1IJ50, 1IJ57GQxx, , 1IJ76 OHIP: 410, 412, 413, R742, R743, Z434, G298 |
| Ontario Congestive heart failure dataset | Cumulative ICES-created disease-specific database from 1988 to 2016 using DAD, SDS, and OHIP:  From -DAD, SDS, one of the following:  ICD-9: 491, 492, 496ICD-10: J41, J42, J43, J44  OR  From OHIP, 2 of the following within a one-year period:  Diagnostic code: 491, 492, 496 |
| Hypertension | Cumulative ICES-created disease-specific database from 1988 to 2016 using DAD and OHIP:  2 physician billing claims (OHIP) or 1 hospital discharge (DAD) with a diagnosis of hypertension in a 2-year period that had the following diagnostic codes: I10.x, I11.x, I12.x, I13.x, or I15.x |
| Inflammatory Bowel Disease | Cumulative ICES-created disease-specific database using DAD, SDS, and OHIP:  ≥1 Hospitalization (DAD,SDS) OR physician visit (OHIP) for the following:  ICD10: K500, K501, K508-K515, K518, K519, M074, M075, M091, M092, K52  OHIP 555, 556, 564 |
| Stroke | Any of the following in the 5 years prior to index date:  ≥1 Hospitalization (DAD) with:  ICD-10: G45, G450, G451, G452 G453 G458 G459, H34.0 H34.1, I60 , I600, I601 , I602 , I603 , I604 , I605, I606 , I607 , I609,I61 ,I63 I630 I631 I632 I633 I634 I635 I638 I639, I64 |
| Stomach or intestinal ulcers | Any of the following in the 5 years prior to index date:  ≥1 Hospitalization (DAD) or physician visit (OHIP) for the following:  ICD-9: 531.0-531.91, 532.0-532.91, 5330-53390, 5340-53491, 56982  ICD-10: K25, K26, K28, K633 |

DAD: Discharge Abstract Database

DXCODE: Diagnostic code

ICD-9: International Classification of Disease, version 9

ICD-10: International Classification of Disease, version 10

ODB: Ontario Drug Benefit Plan

OHIP: Ontario Health Insurance Plan

OMHRS: Ontario Mental Health Reporting System

NACRS: National Ambulatory Care Reporting System

SDS: Same Day Surgery (derived from NACRS)
