# Supplementary material for: Effect of socio-demographic and health factors on the association between multimorbidity and acute care service use: population-based survey linked to health administrative data
Source: BMC Health Serv Res. 2021 Jan 13;21:62. doi: 10.1186/s12913-020-06032-5 (PMC7805153; doi:10.1186/s12913-020-06032-5)
Supplement: Supplementary file 3 — Additional file 3. Canadian Community Health Study Covariates, Operationalization, and Percent Missing. [file 12913_2020_6032_MOESM3_ESM.docx]

**Additional File 3: Canadian Community Health Study Covariates, Operationalization, and Percent Missing**

| **Domain** | **Covariate** | **Operationalization** | **% Missing** |
| --- | --- | --- | --- |
| Demographic | Age | Age at time of CCHS completion | 0.00 |
|  | Sex | Identifies patient sex (Male, Female) | 0.00 |
|  | Immigration status | Ascertains if the participant is an immigrant (yes, no) | 0.25 |
|  | Living arrangement | Identifies the relationship of the participant to all others living in their home. All individuals living with the participant regardless of the relation were grouped together to categorize the variable as living with someone vs. living alone. | 0.18 |
|  | Rurality | Rurality was obtained through linkage of the postal code of the participant at time of CCHS survey completion in the Registered Persons Database (RPDB) to the Postal Code Conversion File (PCCF). Based on Statistics Canada Census data, rurality is based on community size: ( <10,000 vs. ≥10,000) | 0.00 |
| Socioeconomic | Household level of education | Identifies the highest level of education of any member of the household (Post-Secondary Degree/Diploma, Secondary School Diploma, No Diploma) | 3.22 |
|  | Household income | The total household income of the participant from all sources (Less than $30,000,$30,000-$79,000, $80,000 or more) | 7.56 |
| Health Status | Self-reported physical health | Indicates the participants perceived physical health status (Very good/Excellent, Good, Poor/Fair) | 0.11 |
|  | Self-reported mental health | Indicates the participants perceived mental health status (Very good/Excellent, Good, Poor/Fair) | 2.25 |
|  | Activities of daily living (ADL) | Identified if patients need help with any of the following activities of daily living due to any physical condition, mental condition or health problem: preparing meals, getting to appointments or running errands, doing normal everyday housework, personal care such as washing, dressing, eating or taking medication, moving about inside the house, looking after your personal finances such as making bank transactions or paying bills. (Needing help with any of the tasks vs. Does not need help) | 0.07 |
