# Supplementary material for: Effect of socio-demographic and health factors on the association between multimorbidity and acute care service use: population-based survey linked to health administrative data
Source: BMC Health Serv Res. 2021 Jan 13;21:62. doi: 10.1186/s12913-020-06032-5 (PMC7805153; doi:10.1186/s12913-020-06032-5)
Supplement: Supplementary file 4 — Additional file 4. Remaining Stratified Figures for Odds of Hospitalization. [file 12913_2020_6032_MOESM4_ESM.docx]

**Additional File 5: Remaining Stratified Figures for Odds of Hospitalization**


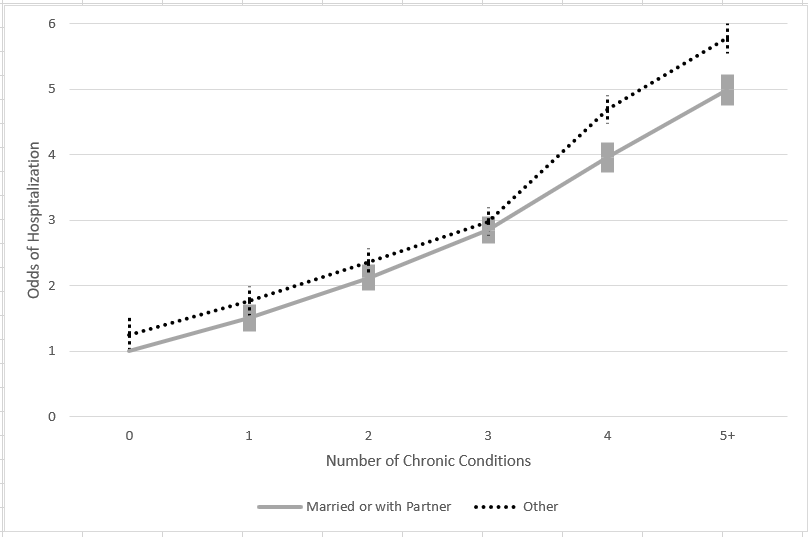


**Figure 4a: Odds of Hospitalization by Marital Status and Number of Chronic Conditions**


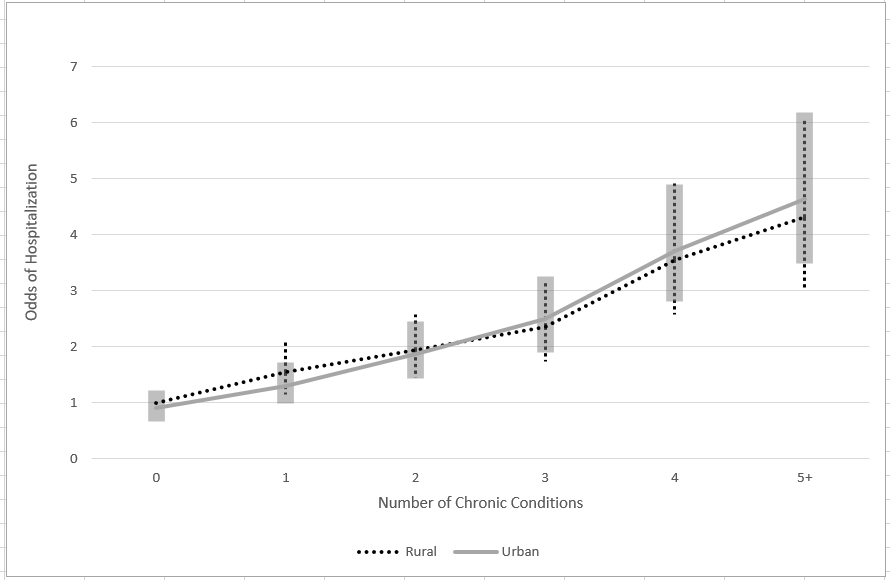


**Figure 4b: Odds of Hospitalizations by Rural Residence and Number of Chronic Conditions**

**
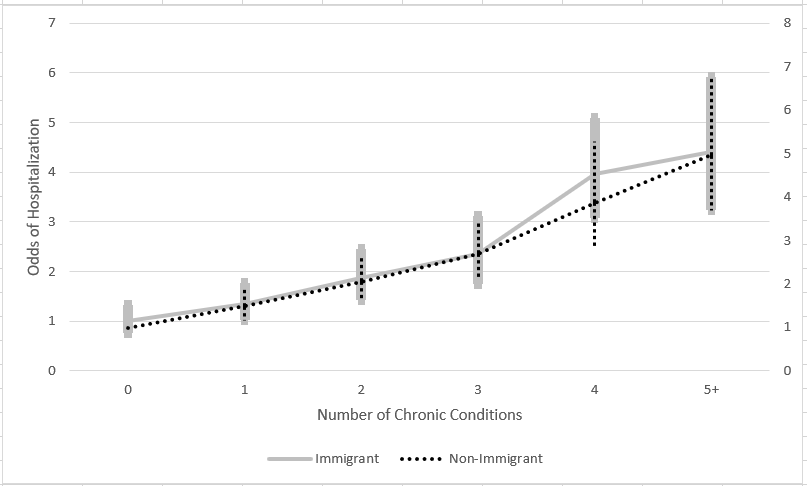
**

**Figure 4c: Odds of Hospitalizations by Immigrant Status and Number of Chronic Conditions**

**
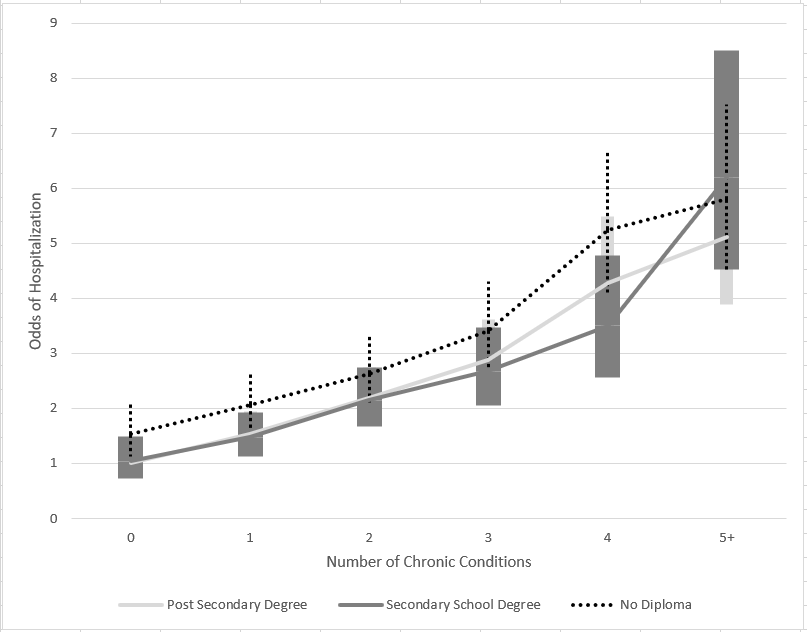
**

**Figure 4d: Odds of Hospitalizations by Education Level and Number of Chronic Conditions**

**
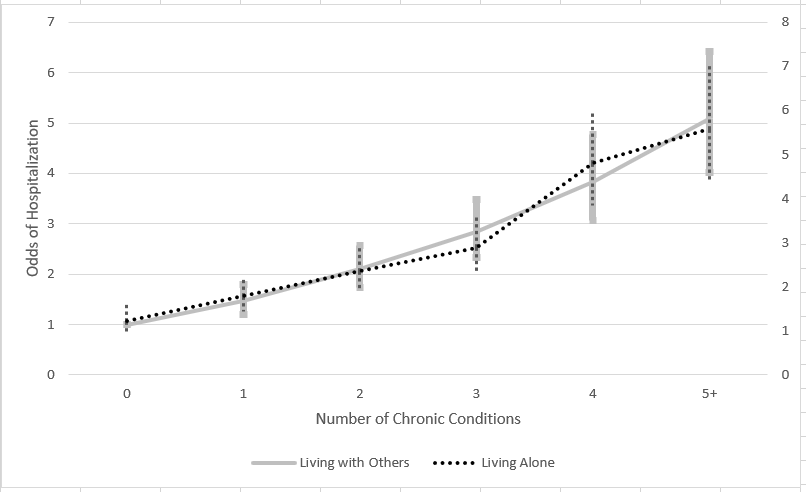
**

**Figure 4e: Odds of Hospitalizations by Household Composition and Number of Chronic Conditions**
