# Supplementary material for: Effect of socio-demographic and health factors on the association between multimorbidity and acute care service use: population-based survey linked to health administrative data
Source: BMC Health Serv Res. 2021 Jan 13;21:62. doi: 10.1186/s12913-020-06032-5 (PMC7805153; doi:10.1186/s12913-020-06032-5)
Supplement: Supplementary file 5 — Additional file 5. Stratified Figures for Odds of Emergency Dept Visit. [file 12913_2020_6032_MOESM5_ESM.docx]

**Additional File 5: Stratified Figures for Odds of Emergency Dept Visit**

**
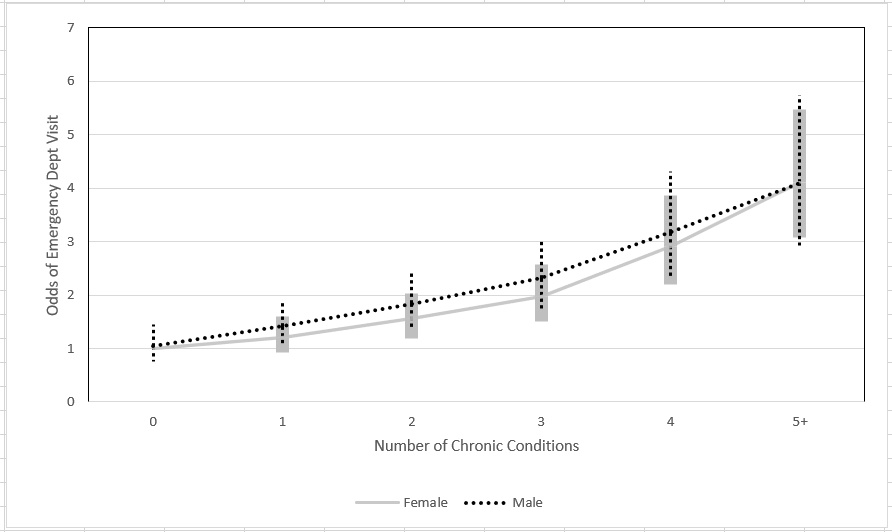
**

**Figure 5a: Odds of Emergency Department Visits by Sex and Number of Chronic Conditions**

**
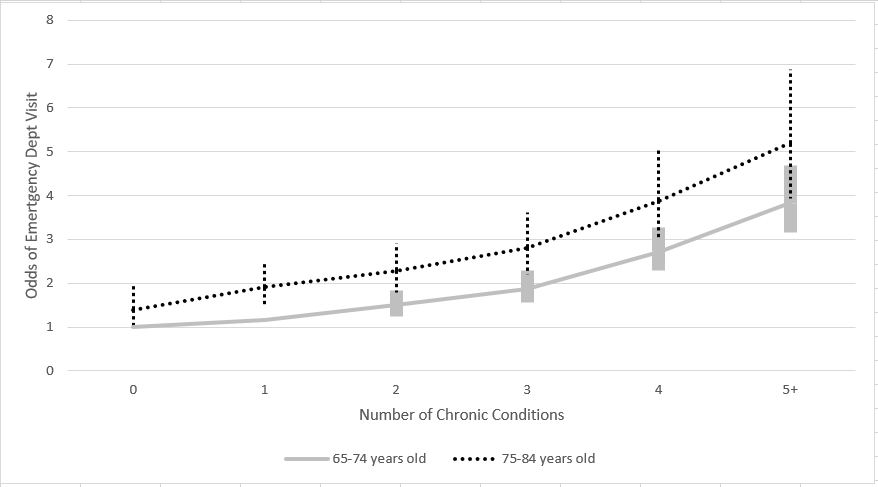
**

**Figure 5b: Odds of Emergency Department Visits by Age and Number of Chronic Conditions**

**
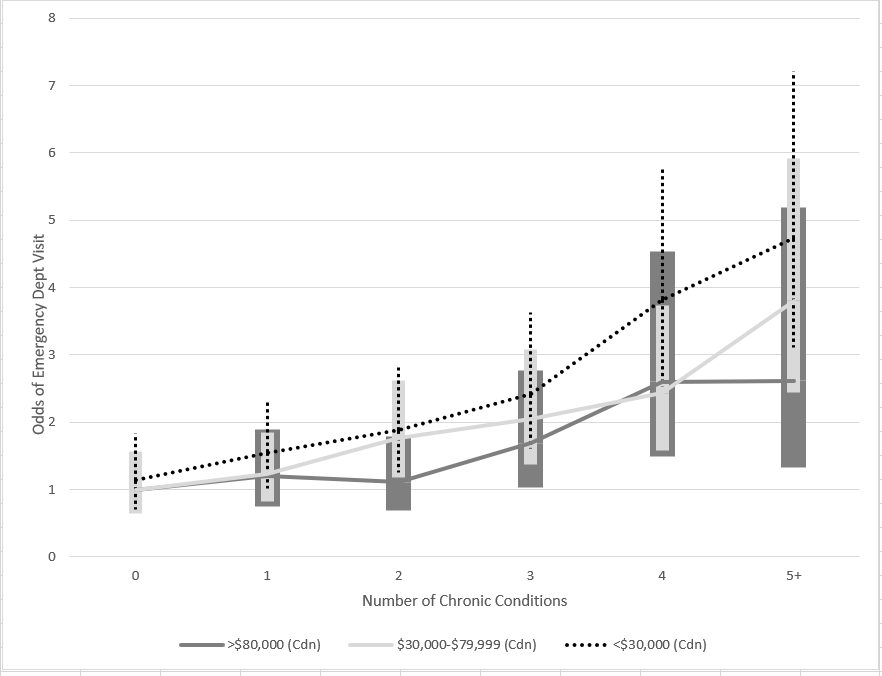
**

**Figure 5c: Odds of Emergency Department Visits by Household Income and Number of Chronic Conditions**

**
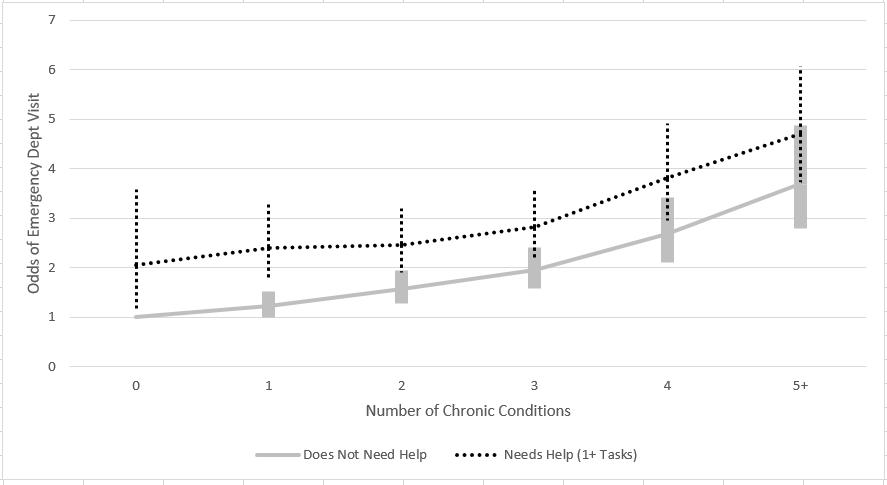
**

**Figure 5d: Odds of Emergency Department Visits by Needs Help and Number of Chronic Conditions**

**
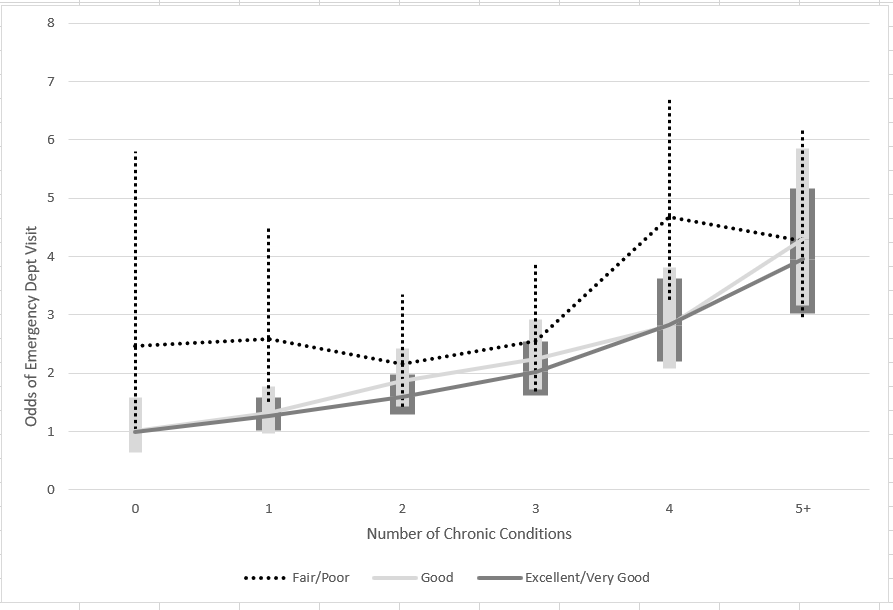
**

**Figure 5e: Odds of Emergency Department Visits Self-Perceived Mental Health by Number of Chronic Conditions**

**
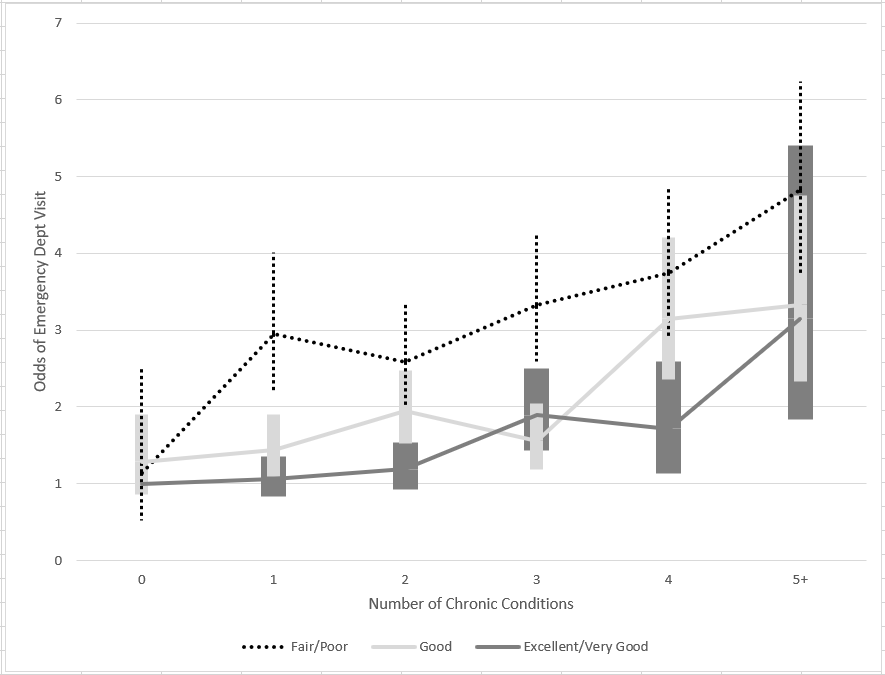
**

**Figure 5f: Odds of Emergency Department Visits by Self-Perceived Physical Health and Number of Chronic Conditions**

**
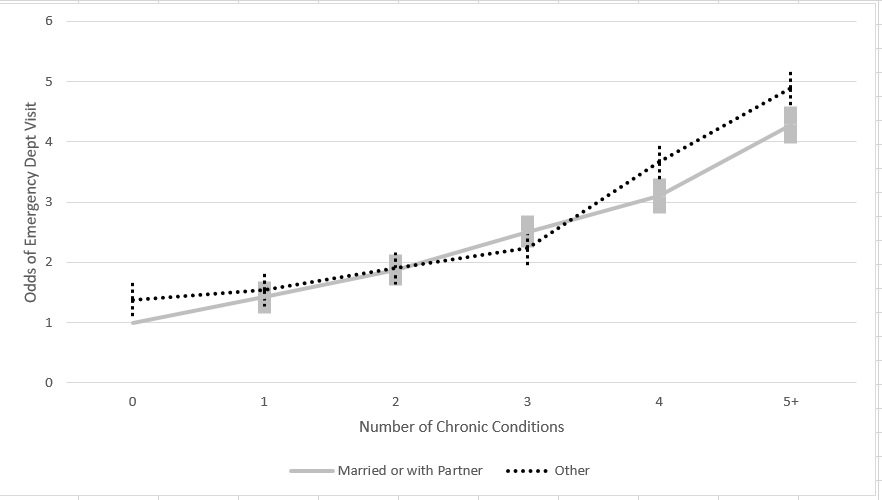
**

**Figure 5g: Odds of Emergency Department Visits by Marital Status and Number of Chronic Conditions**

**
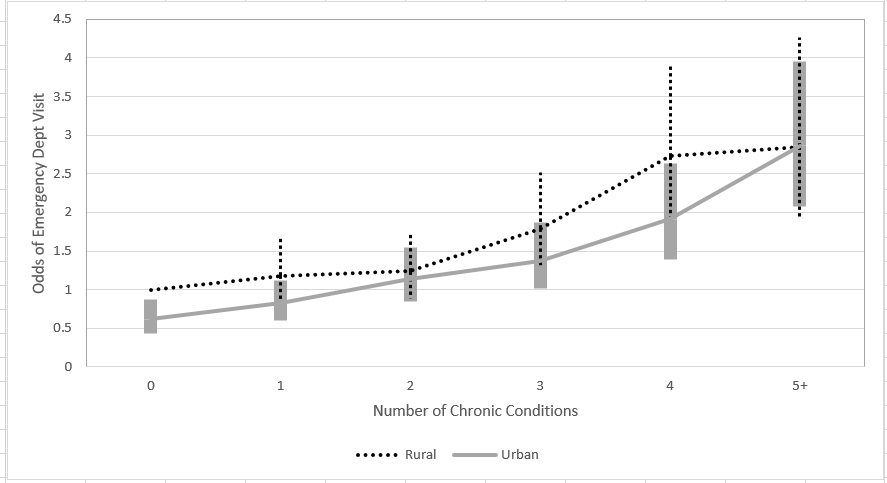
**

**Figure 5h: Odds of Emergency Department Visits by Rural Residency and Number of Chronic Conditions**

**
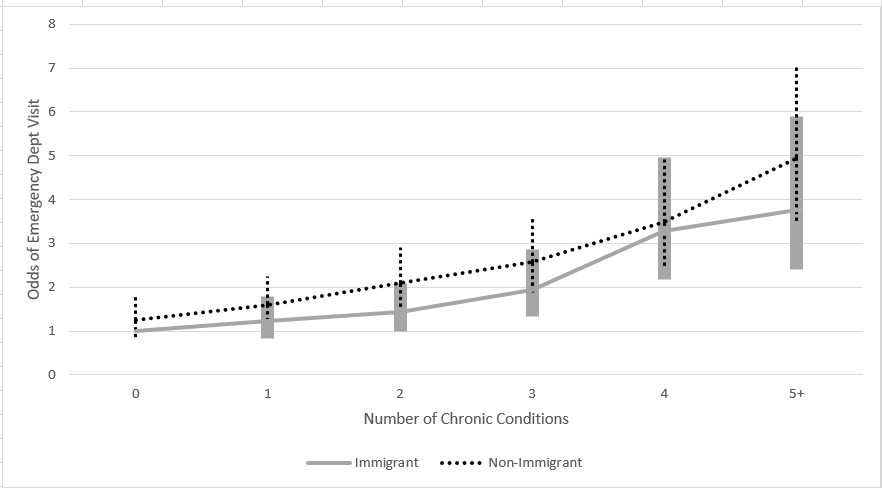
**

**Figure 5i: Odds of Emergency Department Visits by Immigrant Status and Number of Chronic Conditions**

**
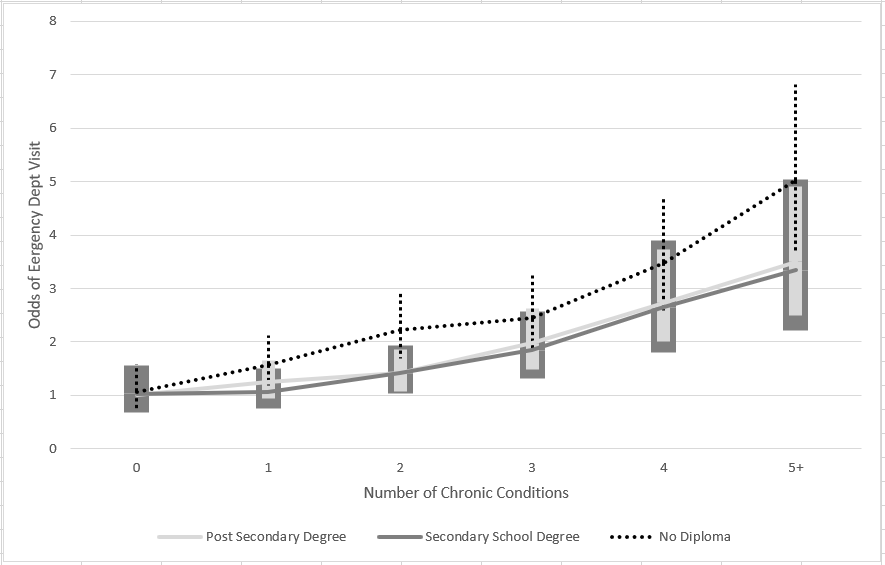
**

**Figure 5j: Odds of Emergency Department Visits by Education Level and Number of Chronic Conditions**

**
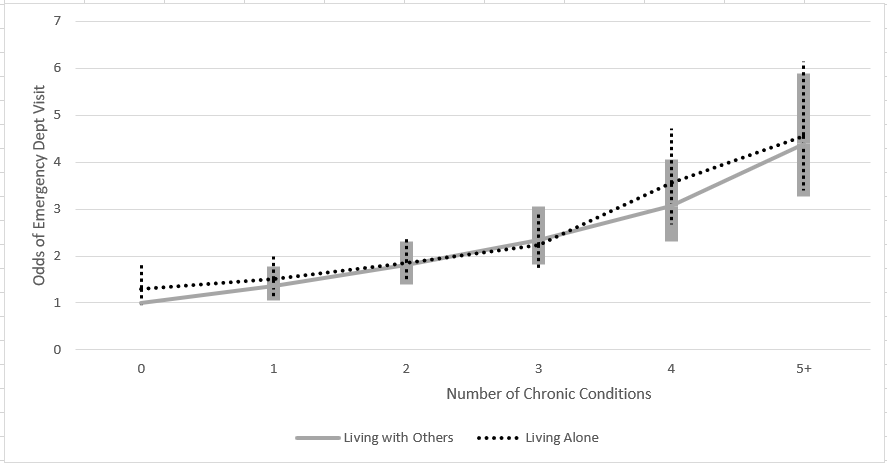
**

**Figure 5k: Odds of Emergency Department Visits by Household Composition and Number of Chronic Conditions**
