# Supplementary material for: Effect of socio-demographic and health factors on the association between multimorbidity and acute care service use: population-based survey linked to health administrative data
Source: BMC Health Serv Res. 2021 Jan 13;21:62. doi: 10.1186/s12913-020-06032-5 (PMC7805153; doi:10.1186/s12913-020-06032-5)
Supplement: Supplementary file 6 — Additional file 6. Interaction Effects Analysis for Emergency Department Visits – Multiple Imputation Results. [file 12913_2020_6032_MOESM6_ESM.docx]

**Additional File 6: : Interaction Effects Analysis for Emergency Department Visits – Multiple Imputation Results**

| **Sex, Age**  **Group** | **MM^a^ Group** | **Correlate Group** | **Overall N (% with 1+Emergency Dept. Visit)** | **OR (95% CI)^b^** | **Inter.^c^**  **(p-value)** |
| --- | --- | --- | --- | --- | --- |
| **Interaction #1: MM x Income** | | | | | |
| Female, 65-74 | 0-1 | >$80k | 640 (4) | Ref | **0.04** |
|  |  | $30k-$79.9k | 1950 (4) | 0.91 (0.59, 1.41) |  |
|  |  | <$30k | 1286 (3) | 0.61 (0.37, 1.01) |  |
|  | 2-3 | >$80k | 507 (4) | Ref |  |
|  |  | $30k-$79.9k | 1955 (5) | 1.14 (0.71, 1,83) |  |
|  |  | <$30k | 1672 (5) | 1.22 (0.76,1.98) |  |
|  | 4+ | >$80k | 108 (7) | Ref |  |
|  |  | $30k-$79.9k | 563 (8) | 1.14 (0.52 2.48) |  |
|  |  | <$30k | 749 (12) | 1.66 (0.78, 3.54) |  |
| Female, 75-84 | 0-1 | >$80k | 210 (8) | Ref | 0.29 |
|  |  | $30k-$79.9k | 937 (5) | 0.83 (0.42, 1.60) |  |
|  |  | <$30k | 962 (6) | 1.10 (0.58, 2.08) |  |
|  | 2-3 | >$80k | 285 (4) | Ref |  |
|  |  | **$30k-$79.9k** | **1421 (8)** | **2.05 (1.09, 3.86)** |  |
|  |  | **<$30k** | **1719 (9)** | **2.49 (1.33, 4.64)** |  |
|  | 4+ | >$80k | 102 (13) | Ref |  |
|  |  | $30k-$79.9k | 429 (11) | 0.86 (0.45, 1.66) |  |
|  |  | <$30k | 773 (15) | 1.22 (0.66, 2.66) |  |
| Male, 65-74 | 0-1 | >$80k | 911 (3) | Ref | 0.22 |
|  |  | $30k-$79.9k | 1805 (4) | 1.14 (0.73, 1.77) |  |
|  |  | **<$30k** | **804 (6)** | **1.93 (1.21, 3.09)** |  |
|  | 2-3 | >$80k | 686 (5) | Ref |  |
|  |  | **$30k-$79.9k** | **1752 (7)** | **1.58 (1.05, 2.37)** |  |
|  |  | <$30k | 798 (7) | 1.56 (0.99, 2.45) |  |
|  | 4+ | >$80k | 107 (6) | Ref |  |
|  |  | $30k-$79.9k | 407 (10) | 1.78 (0.74, 4.33) |  |
|  |  | **<$30k** | **279 (14)** | **2.74 (1.12, 6.66)** |  |
| Male, 75-84 | 0-1 | >$80k | 253 (6) | Ref | 0.92 |
|  |  | $30k-$79.9k | 866 (6) | 0.95 (0.52, 1.72) |  |
|  |  | <$30k | 483 (8) | 1.47 (0.808, 2.71) |  |
|  | 2-3 | >$80k | 350 (7) | Ref |  |
|  |  | $30k-$79.9k | 1238 (8) | 1.20 (0.77 1.90) |  |
|  |  | <$30k | 616 (10) | 1.51 (0.93, 2.44) |  |
|  | 4+ | >$80k | 97 (11) | Ref |  |
|  |  | $30k-$79.9k | 400 (12) | 1.09 (0.54, 2.19) |  |
|  |  | <$30k | 241 (17) | 1.65 (0.81, 3.36) |  |
|  | | | | | |
| Female, 65-74 | 0-1 | Excellent | 3192 (4) | Ref | 0.43 |
|  |  | Good | 627 (4) | 0.97 (0.61, 1.55) |  |
|  |  | Fair/Poor | 57 (<11)^d^ | 2.02 (0.72, 5.67) |  |
|  | 2-3 | Excellent | 3040 (5) | Ref |  |
|  |  | Good | 900 (6) | 1.26 (0.91, 1.76) |  |
|  |  | **Fair/Poor** | **194 (9)** | **2.02 (1.19, 3.42)** |  |
|  | 4+ | Excellent | 828 (10) | Ref |  |
|  |  | Good | 415 (8) | 0.81 (0.53, 1.22) |  |
|  |  | Fair/Poor | 177 (13) | 1.31 (0.80 2.14) |  |
| Female, 75-84 | 0-1 | Excellent | 1633 (6) | Ref | 0.18 |
|  |  | Good | 435 (5) | 0.90 (0.56, 1.46) |  |
|  |  | Fair/Poor | 41 (<15)^d^ | 1.83 (0.64, 5.25) |  |
|  | 2-3 | Excellent | 2404 (8) | Ref |  |
|  |  | Good | 864 (8) | 0.94 (0.71, 1.26) |  |
|  |  | Fair/Poor | 157 (7) | 0.84 (0.44, 1.58) |  |
|  | 4+ | Excellent | 740 (11) | Ref |  |
|  |  | Good | 838 (15) | 1.38 (0.96, 1.97) |  |
|  |  | **Fair/Poor** | **181 (19)** | **1.85 (1.20, 2.85)** |  |
| Male, 65-74 | 0-1 | Excellent | 2792 (4) | Ref | 0.19 |
|  |  | Good | 643 (4) | 1.02 (0.65, 1.58) |  |
|  |  | **Fair/Poor** | **85 (12)** | **3.47 (1.68, 6.66)** |  |
|  | 2-3 | Excellent | 2257 (6) | Ref |  |
|  |  | Good | 805 (7) | 1.10 (0.79, 1.52) |  |
|  |  | Fair/Poor | 174 (9) | 1.44 (0.82, 2.51) |  |
|  | 4+ | Excellent | 444 (10) | Ref |  |
|  |  | Good | 242 (11) | 1.11 (0.67, 1.84) |  |
|  |  | Fair/Poor | 107 (11) | 1.12 (0.57, 2.20) |  |
| Male, 75-84 | 0-1 | Excellent | 1173 (6) | Ref | 0.82 |
|  |  | Good | 371 (7) | 1.10 (0.69, 1.75) |  |
|  |  | Fair/Poor | 58 (< 11)^d^ | 1.08 (0.38, 3.08) |  |
|  | 2-3 | Excellent | 1459 (8) | Ref |  |
|  |  | Good | 597 (10) | 1.32 (0.95, 1.82) |  |
|  |  | Fair/Poor | 148 (9) | 1.19 (0.66, 2.12) |  |
|  | 4+ | Excellent | 407 (14) | Ref |  |
|  |  | Good | 208 (13) | 0.92 (0.56, 1.50) |  |
|  |  | Fair/Poor | 123 (15) | 1.05 (0.59, 1.87) |  |

| **Interaction #2: MM x Instrumental Activities of Daily Living** | | | | | |
| --- | --- | --- | --- | --- | --- |
| Female, 65-74 | 0-1 | Does not need help | 3575 (3) | Ref | 0.72 |
|  |  | **Needs help** | **301 (7)** | **2.03 (1.25, 3,31)** |  |
|  | 2-3 | Does not need help | 3372 (4) | Ref |  |
|  |  | **Needs help** | **762 (7)** | **1.70 (1.21, 2.28)** |  |
|  | 4+ | Does not need help | 833 (8) | Ref |  |
|  |  | **Needs help** | **587 (13)** | **1.60 (1.13, 2.26)** |  |
| Female, 75-84 | 0-1 | Does not need help | 1750 (5) | Ref | 0.75 |
|  |  | **Needs help** | **359 (8)** | **1.66 (1.07, 2.57)** |  |
|  | 2-3 | Does not need help | 2235 (7) | Ref |  |
|  |  | **Needs help** | **1190 (10)** | **1.50 (1.17, 1.93)** |  |
|  | 4+ | Does not need help | 526 (12) | Ref |  |
|  |  | Needs help | 778 (15) | 1.35 (0.97, 1.88) |  |
| Male, 65-74 | 0-1 | Does not need help | 3373, (4) | Ref | 0.05 |
|  |  | **Needs help** | **147 (9)** | **2.44 (1.34,4.43)** |  |
|  | 2-3 | Does not need help | 2895 (6) | Ref |  |
|  |  | Needs help | 341 (7) | 1.12 (0.72, 1.73) |  |
|  | 4+ | Does not need help | 575 (11) | Ref |  |
|  |  | Needs help | 218 (11) | 0.99 (0.60, 1.65) |  |
| Male, 75-84 | 0-1 | Does not need help | 1430 (6) | Ref | 0.70 |
|  |  | **Needs help** | **172 (10)** | **1.80 (1.06, 3.07)** |  |
|  | 2-3 | Does not need help | 1752 (8) | Ref |  |
|  |  | **Needs help** | **452 (12)** | **1.52 (1.08, 2.12)** |  |
|  | 4+ | Does not need help | 416 (12) | Ref |  |
|  |  | Needs help | 322 (16) | 1.35 (0.89, 2.05) |  |

^a^ MM = multimorbidity

^b^ OR (95% CI) = Odds Ratio of 1+ Hospitalization (95% Confidence Interval)

^c^ Inter. = Interaction

^d^ (%) with 1+ hospitalization rounded upward to avoid reporting of small cell size.
