# Supplementary material for: Effect of socio-demographic and health factors on the association between multimorbidity and acute care service use: population-based survey linked to health administrative data
Source: BMC Health Serv Res. 2021 Jan 13;21:62. doi: 10.1186/s12913-020-06032-5 (PMC7805153; doi:10.1186/s12913-020-06032-5)
Supplement: Supplementary file 7 — Additional file 7. Odds Ratios and Incidence Rates of Emergency Department Visits - Multiple Imputation Results. [file 12913_2020_6032_MOESM7_ESM.docx]

**Additional File 7: Odds Ratios and Incidence Rates of Emergency Department Visits - Multiple Imputation Results**

| **Variable** | **OR [95%CI]^a^** | **IR [95%CI]^a^** |
| --- | --- | --- |
| **Chronic Conditions** |  |  |
| 0-1 | - | - |
| 2-3 | **1.21 [1.08, 1.36]** | **1.30 [1.14, 1.48]** |
| 4+ | **1.75 [1.51, 2.02]** | **1.73 [1.45, 2.06]** |
| **Age** |  |  |
| 65-74 | - | - |
| 75-84 | **1.43 [1.30, 1.58]** | **1.38 [1.23, 1.54]** |
| **Sex** |  |  |
| Female | - | - |
| Male | **1.16 [1.05, 1.29]** | **1.24 [1.11, 1.40]** |
| **Immigrant Status** |  |  |
| Immigrant | - | - |
| Non-Immigrant | **1.35 [1.20, 1.51]** | **1.41 [1.24, 1.60]** |
| **Education** |  |  |
| Post-secondary degree | - | - |
| Secondary school degree | 0.91 [0.80, 1.04] | 1.00 [0.86, 1.16] |
| No diploma | **1.12 [1.00, 1.26]** | **1.14 [1.00, 1.30]** |
| **Household Income** |  |  |
| Over $80,000 | - | - |
| $30,000 to $79,999 | 1.12 [0.95, 1.32] | 1.16 [0.96, 1.39] |
| Under $30,000 | **1.29 [1.07, 1.55]** | **1.36 [1.11, 1.67]** |
| **Living Arrangement** |  |  |
| Living with others | - | - |
| Living alone | 0.98 [0.88, 1.09] | 0.97 [0.86, 1.10] |
| **Geography** |  |  |
| Urban | - | - |
| Rural | **1.26 [1.13, 1.40]** | **1.33 [1.18, 1.51]** |
| **Instrumental Activities of Daily Living (IADLs)** |  |  |
| Does not need help with basic tasks | - | - |
| Needs help with basic tasks | **1.26 [1.12, 1.41]** | **1.23 [1.07, 1.42]** |
| **Self-Perceived Physical Health** |  |  |
| Excellent/Very Good | - | - |
| Good | **1.33 [1.17, 1.51]** | **1.45 [1.26, 1.66]** |
| Fair or Poor | **1.91 [1.66, 2.19]** | **2.03 [1.73, 2.38]** |
| **Self-Perceived Mental Health** |  |  |
| Excellent | - | - |
| Good | 0.93 [0.83, 1.04] | 0.93 [0.81, 1.06] |
| Fair or Poor | 1.02 [0.84, 1.23] | 0.97 [0.76, 1.23] |

^a^ OR [95% CI] = Odds Ratio [95% Confidence Interval], IR [95%CI] = Incidence Rate [95% Confidence Interval]
